# Alterations in Semen Quality and Immune‐Related Factors in Men with Infertility who Recovered from COVID‐19

**DOI:** 10.1002/mco2.70179

**Published:** 2025-04-24

**Authors:** Ying Zhang, Feiyin Zhu, Zhe Zhang, Jing Wang, Tianyi Liao, Yu Xi, Defeng Liu, Haitao Zhang, Haocheng Lin, Jiaming Mao, Wenhao Tang, Lianming Zhao, Peng Yuan, Liying Yan, Qiang Liu, Kai Hong, Jie Qiao

**Affiliations:** ^1^ Department of Obstetrics and Gynecology Center for Reproductive Medicine Peking University Third Hospital Beijing China; ^2^ State Key Laboratory of Female Fertility Promotion National Clinical Research Center for Obstetrics and Gynecology Key Laboratory of Assisted Reproduction (Peking University) Ministry of Education Beijing Key Laboratory of Reproductive Endocrinology and Assisted Reproductive Technology Peking University Third Hospital Beijing China; ^3^ Peking‐Tsinghua Center for Life Sciences Peking University Beijing China; ^4^ Department of Urology Peking University Third Hospital Beijing China

**Keywords:** male infertility, semen quality, COVID‐19, fever, immune response

## Abstract

The emergence of coronavirus disease 2019 (COVID‐19) has triggered research into its impact on male reproductive health. However, studies exploring the effects of severe acute respiratory syndrome coronavirus 2 (SARS‐CoV‐2) on semen quality in infertile men remain limited. Herein, we enrolled 781 male infertile patients who recovered from COVID‐19 and analyzed their semen and blood samples collected at different time points. We found that SARS‐CoV‐2 RNA was undetectable in semen samples. Compared with pre‐COVID‐19 status, total sperm count, sperm concentration, vitality, motility, and percentage of sperm cells with normal morphology decreased significantly in the first month post‐COVID‐19. However, these alterations were reversed in the third month. Furthermore, seminal plasma samples exhibited reduced proinflammatory cytokine levels and notable changes in amino acid, nucleic acid, and carbohydrate metabolism by the third month compared with those in the first month. By contrast, no significant alterations in reproductive hormone levels were found. Vitality, progressive motility, and total motility negatively correlated with body temperature when it was above 38°C. In conclusion, semen quality initially decreases post‐COVID‐19 but reverses after approximately 3 months, with a decline related to inflammatory and fever. These findings may provide guidance to infertile male patients who need assisted reproductive technology.

## Graphical abstract

Semen quality initially decreases post‐COVID‐19 but reverses after approximately 3 months in male with infertility, and the decreased semen quality was related to inflammatory reactions and fever

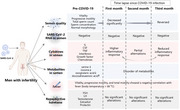

## 1 Introduction

Coronavirus disease 2019 (COVID‐19), caused by severe acute respiratory syndrome coronavirus 2 (SARS‐CoV‐2), has profoundly impacted health and healthcare systems worldwide [1]. As researchers continue to investigate the virus and its influence on the human body, emerging evidence indicates that COVID‐19 may affect male fertility and semen quality [2].

Although COVID‐19 was originally considered a respiratory illness, it can damage several organ systems [3, 4]. Since the outbreak of the COVID‐19 pandemic, multiple organ manifestations, including respiratory, cardiovascular, and gastrointestinal, have been reported [5]. A study found that 70% of the 201 participants examined exhibited impairments in one or more organs, including the heart, lungs, liver, kidneys, pancreas, and spleen, with 29% experiencing extensive damage [6]. Chronic respiratory conditions associated with COVID‐19, such as pulmonary fibrosis, were highlighted in a study by Han et al [7]. Cardiovascular disruptions include endothelial dysfunction, deep vein thrombosis, pulmonary embolism, and hemorrhage [8, 9, 10]. Gastrointestinal symptoms including nausea, abdominal pain, decreased appetite, and constipation, with changes in gut microbiota potentially worsening inflammation [11, 12, 13]. Additionally, patients with COVID‐19 frequently have reproductive challenges. Evidence suggests a reduction in ovarian reserves and disruptions to the reproductive endocrine function in affected individuals [14]. One study proposes that SARS‐CoV‐2 may impact ovarian follicles, as shown by experiments with granulosa and cumulus cells [15]. Furthermore, an increased risk of maternal morbidity and mortality has been documented [16], alongside higher incidence rates of preterm birth and perinatal mortality linked to SARS‐CoV‐2 infection during pregnancy [17, 18, 19].

Studies have reported on the impact of COVID‐19 on male fertility. Nie et al. [20] found that patients with testicular impairment have decreased numbers of Leydig cells, disrupted cholesterol production, and compromised sperm motility. Similarly, Shcherbitskaia et al. [21]. and Aksak et al. [22] revealed that SARS‐CoV‐2 adversely affects semen quality and disrupts multiple biochemical processes in semen and spermatogenic cells, resulting in impaired male fertility. However, these studies are limited by factors, such as a modest sample size, age disparities between patients and controls, and limited generalizability, as only young, healthy men and a few individuals with fertility problems were enrolled.

Initial studies indicated that SARS‐CoV‐2 may interfere with spermatogenesis by directly targeting testicular cells, which are equipped with the angiotensin‐converting enzyme 2 (ACE2) receptor to which the spike glycoprotein of the virus binds for cellular entry [23, 24]. Testosterone and androgen receptors, which are coregulators of the ACE2 receptor, may facilitate the internalization of SARS‐CoV‐2 into the male reproductive system [25]. Additionally, the inflammatory response and cytokine release associated with SARS‐CoV‐2 infection may negatively affect the sperm production and function [26]. Infertility is a significant reproductive health concern and is estimated to affect approximately 15% of couples globally [27]. Male infertility, which accounts for approximately half of all infertility cases, typically presents as a reduction in sperm count (azoospermia or oligozoospermia), diminished sperm motility (asthenozoospermia), or a higher proportion of morphologically abnormal sperm (teratozoospermia) [28]. In addition, data regarding the effects of SARS‐CoV‐2 on semen quality in men with fertility issues remain insufficient.

To further investigate the impact of COVID‐19 on semen quality in male with infertility, we conducted a study involving 781 patients who were diagnosed with infertility. This study, with the largest sample size, focused on the alteration of semen quality, cytokines, and metabolic levels in infertile male patients after COVID‐19 infection. The findings provide insights into the underlying causes and may help refine therapeutic strategies.

## 2 Results

### 2.1 Alterations in the Semen Parameters Post‐COVID‐19

We initially investigated the effects of post‐COVID‐19 infection on semen parameters. Semen samples from 1294 infertile men were grouped according to different time points post‐COVID‐19: 15.69% (*n* = 203) in the first month (0–30 days group), 40.11% (*n* = 519) in the second month (31–60 days group), 19.17% (*n* = 248) in the third month (61–90 days group), and 6.96% (*n* = 90) in ≥91 days group; data of 18.08% (*n* = 234) samples in the control group (pre‐COVID‐19 group) were obtained before COVID‐19 infection. The mean ages of the participants in each group were 34.17 ± 4.09, 33.70 ± 4.66, 33.48 ± 4.66, 33.21 ± 4.41, and 33.53 ± 4.61, respectively, with no significant age differences across the groups. Of all examined samples, 20.25% (262 out of 1294) were oligozoospermic, 66.46% (860 out of 1294) were asthenozoospermic, and 67.77% (877 out of 1294) were teratozoospermic. Semen quality parameters were analyzed and compared across the different groups. We found that compared with those in the pre‐COVID‐19 group, several sperm parameters were significantly altered post‐COVID‐19, such as sperm concentration, vitality, total motility, progressive motility, and sperm cells with normal morphology. These findings suggest that COVID‐19 has a significant negative effect on spermatogenesis (Table 1). Notably, the alterations in sperm parameters were largely reversed in the third month (61–90 days) post‐COVID‐19 compared with those pre‐COVID‐19 (Table S1).

**TABLE 1:** Sperm quality in relation to timing at different time points post‐COVID‐19 infection.

		Time lapse since COVID‐19 infection				
Characteristic	Pre‐COVID‐19	0–30 days	31–60 days	61–90 days	≥91 days	*p *value
No. of samples	234 (18.08%)	203 (15.69%)	519 (40.11%)	248 (19.17%)	90 (6.96%)	/
No. of days postinfection	/	21.03 ± 7.41	45.56 ± 8.15	73.68 ± 8.54	106.71 ± 23.09	/
Age, y	34.17 ± 4.09	33.70 ± 4.66	33.48 ± 4.66	33.21 ± 4.41	33.53 ± 4.61	0.0829
Volume, mL	3.38 ± 2.39	3.03 ± 1.31	3.00 ± 1.49	3.16 ± 1.28	2.90 ± 1.25	0.1916
Sperm concentration, million/mL	46.75 ± 35.86	32.22 ± 29.55	38.06 ± 37.13	47.38 ± 42.55	41.04 ± 32.41	<0.0001
Total sperm count, million	141.92 ± 123.72	98.15 ± 85.00	118.33 ± 149.55	136.12 ± 107.07	107.69 ± 81.54	<0.0001
Vitality, %	33.88 ± 18.89	27.29 ± 17.31	32.81 ± 17.67	34.66 ± 18.54	33.87 ± 14.38	<0.0001
Progressive motility, %	28.30 ± 17.93	21.90 ± 15.88	26.96 ± 16.73	28.73 ± 16.94	28.59 ± 14.09	<0.0001
Total motility, %	33.89 ± 18.88	27.34 ± 17.45	32.4 ± 17.65	34.41 ± 18.34	33.94 ± 14.27	<0.0001
Normal morphology, %	2.45 ± 1.21	2.00 ± 1.29	2.23 ± 1.27	2.73 ± 1.51	2.36 ± 1.04	<0.0001
Head defect, %	97.02 ± 9.37	96.97 ± 1.82	96.75 ± 2.27	96.00 ± 2.48	96.60 ± 1.53	<0.0001
Neck defect, %	0.63 ± 0.51	0.59 ± 0.60	0.57 ± 0.54	0.69 ± 0.66	0.57 ± 0.42	0.2528
Tail defect, %	0.55 ± 0.44	0.43 ± 0.53	0.39 ± 0.44	0.50 ± 0.60	0.46 ± 0.35	0.0106

Data are presented as mean ± standard deviation or number (%). The *p* values of outcome events are calculated using Kruskal–Wallis test. “/” means not available.

We further analyzed semen samples from the same participant before COVID‐19 and at various time points post‐COVID‐19. Compared with the sperm parameters before COVID‐19 (Table 2), in the first month post‐COVID‐19 in the same group of individuals (*n* = 75), there was a decrease in sperm concentration (28.70 ± 29.32 vs. 42.88 ± 28.77, million/mL), total sperm count (81.53 ± 72.62 vs. 128.73 ± 91.04, million), vitality (21.99 ± 15.99 vs. 31.50 ± 18.68, %), progressive motility (17.45 ± 14.54 vs. 26.14 ± 17.80, %), total motility (21.96 ± 15.96 vs. 31.50 ± 18.68, %), and sperms cells with normal morphology (1.87 ± 1.33 vs. 2.41 ± 1.13, %). However, there was no significant difference in semen volume (3.20 ± 1.45 vs. 3.23 ± 1.50, mL). A significant decline in sperm quality was observed in the second month post‐COVID‐19 (*n* = 140). However, sperm parameters almost returned to preinfection levels at 61–90 days (*n* = 81) and ≥91 days (*n* = 44) post‐COVID‐19 compared with those in the control samples. Considering that the spermatogenic cycle in humans spans approximately 74 days [29], our findings indicate that the influence of COVID‐19 on spermatogenesis is likely short‐lived, potentially impacting only the first cycle of sperm development that was underway during the infection.

**TABLE 2:** Semen parameters of patients who recovered from COVID‐19.

	First month (*n* = 75)			Second month (*n* = 140)			Third month (*n* = 81)			Over third month (*n* = 44)		
Characteristic	Pre‐COVID‐19	0–30 days	*p *value	Pre‐COVID‐19	31–60 days	*p *value	Pre‐COVID‐19	61–90 days	*p *value	Pre‐COVID‐19	≥91 days	*p *value
Volume (mL)	3.23 ± 1.50	3.20 ± 1.45	0.2937	3.22 ± 1.42	3.04 ± 1.46	0.4204	5.31 ± 10.66	5.38 ± 7.35	0.5972	3.18 ± 1.35	3.11 ± 1.35	0.9645
Sperm concentration (million/mL)	42.88 ± 28.77	28.70 ± 29.32	0.0001	50.51 ± 41.92	36.00 ± 41.00	<0.0001	42.49 ± 35.50	44.13 ± 34.05	0.4821	50.52 ± 43.21	51.35 ± 48.19	0.8579
Total sperm count (million)	128.73 ± 91.04	81.53 ± 72.62	<0.0001	149.91 ± 135.23	135.27 ± 231.21	<0.0001	117.37 ± 95.65	133.83 ± 114.86	0.6871	156.00 ± 181.66	144.83 ± 132.82	0.5284
Vitality (%)	31.50 ± 18.68	21.99 ± 15.99	<0.0001	35.87 ± 18.40	29.03 ± 17.03	<0.0001	34.24 ± 17.47	30.38 ± 17.10	0.1200	32.03 ± 17.62	28.10 ± 16.86	0.2388
Progressive motility (%)	26.14 ± 17.80	17.45 ± 14.54	<0.0001	30.07 ± 17.24	23.60 ± 15.82	<0.0001	28.32 ± 16.84	26.17 ± 21.90	0.1068	25.82 ± 16.69	23.41 ± 16.15	0.5227
Total motility (%)	31.50 ± 18.68	21.96 ± 15.96	<0.0001	35.89 ± 18.34	29.01 ± 16.92	<0.0001	36.12 ± 21.60	32.39 ± 22.99	0.0594	32.08 ± 17.60	28.30 ± 16.71	0.2580
Normal morphology (%)	2.41 ± 1.13	1.87 ± 1.33	0.0037	2.64 ± 1.31	2.25 ± 1.24	0.0186	2.42 ± 1.16	2.13 ± 1.18	0.5194	2.62 ± 1.34	2.55 ± 1.23	0.3988

Data are presented as mean ± standard deviation. The *p* values of outcome events are calculated using paired *t*‐test.

### 2.2 Contagiousness of Semen Post‐COVID‐19

Currently, the presence of SARS‐CoV‐2 in the semen remains controversial. To investigate potential viral transmission through semen post‐COVID‐19, a subset of 96 semen samples was analyzed to detect the presence of SARS‐CoV‐2 RNA. The 96 samples were collected from days 1 to 116 post‐COVID‐19 and divided into four groups: 34.3% (*n* = 33) within 0–30 days post‐COVID‐19, 47.9% (*n* = 46) within 31–60 days post‐COVID‐19, 12.5% (*n* = 12) within 61–90 days post‐COVID‐19, and 5.2% (*n* = 5) ≥91 days post‐COVID‐19. The COVID‐19 polymerase chain reaction (PCR) test results showed no detectable SARS‐CoV‐2 RNA in any of the 96 semen samples obtained from individuals who had recovered from COVID‐19 (Table S2). This suggests that the virus does not persist in a transmissible state in semen following recovery from COVID‐19. The changes in semen parameters described above may be the result of several biological mechanisms acting in concert to disrupt the reproductive system, rather than being caused directly by SARS‐CoV‐2 infection.

### 2.3 Impact of Cytokines on Seminal Plasma Post‐COVID‐19

COVID‐19, being a severe inflammatory disease, inevitably induces changes in the immune system [30, 31]. Several studies have shown that certain inflammatory cytokines, including interferon‐γ (IFN‐γ), interleukin (IL)‐17, and IL‐1β, can directly impair sperm quality. To determine whether the effect of decreased sperm quality was caused by immune response, we examined cytokine levels in seminal plasma samples from the same individuals obtained at different time points post‐COVID‐19. The seminal plasma contains a wide range of cytokines. We specifically examined the levels of 27 representative cytokines: including IL‐1β, IL‐1ra, IL‐2, IL‐4, IL‐5, IL‐6, IL‐7, IL‐8, IL‐9, IL‐10, IL‐12, IL‐13, IL‐15, and IL‐17; colony‐stimulating factor (CSF), including granulocyte‐CSF (G‐CSF) and granulocyte macrophage‐CSF (GM‐CSF); IFN‐γ; tumor necrosis factor‐α (TNF‐α); growth factor, including platelet‐derived growth factor (PDGF), fibroblast growth factor (FGF), and vascular endothelial factor (VEGF); and chemokines, including macrophage inflammatory protein‐10 (IP‐10), eotaxin, macrophage inflammatory protein‐1α (MIP‐1α), MIP‐1β, RANTES, and monocyte chemoattractant protein‐1 (MCP‐1).

**FIGURE 1:**
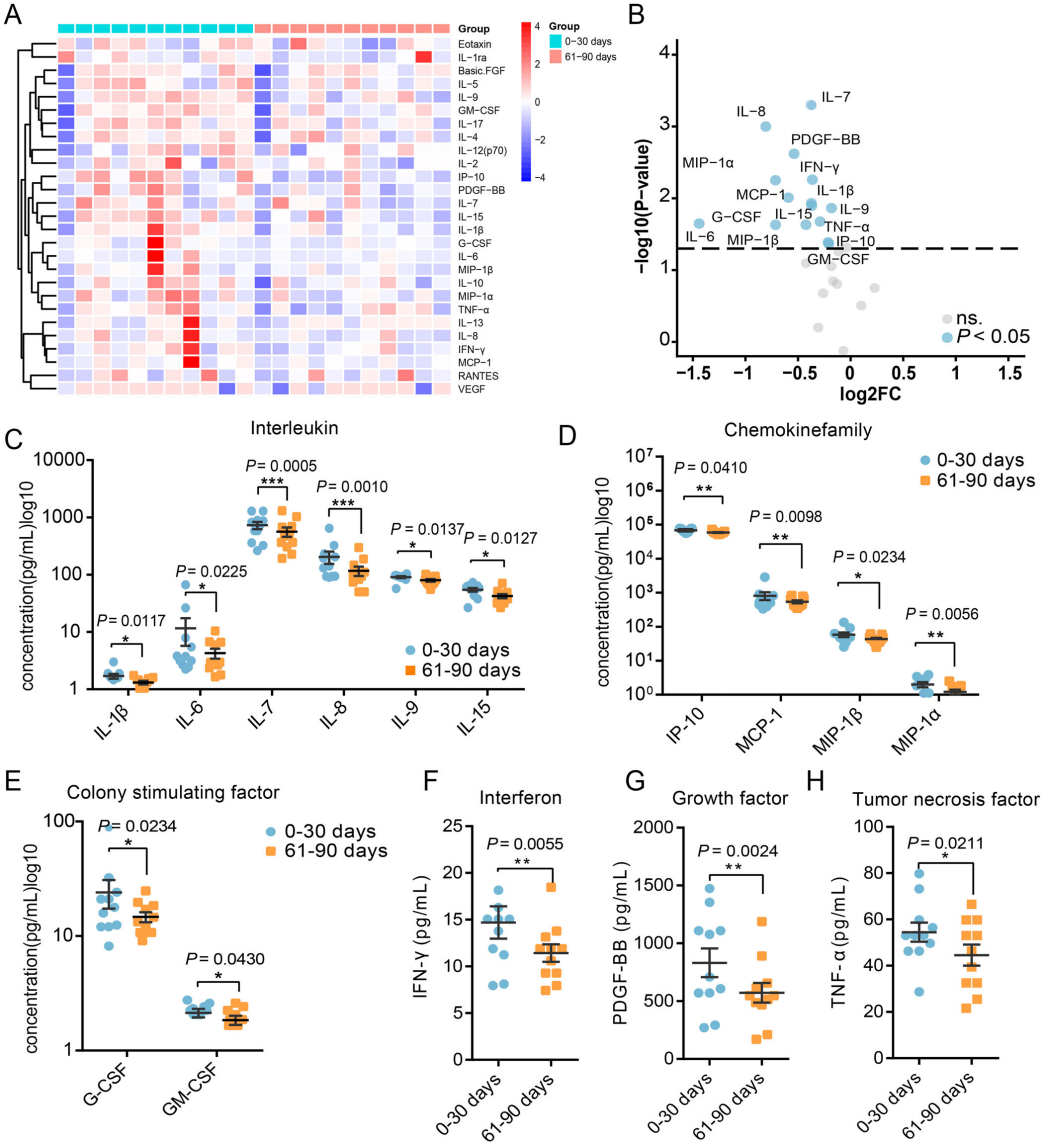
Changes in the inflammatory cytokine levels at indicated time points post‐COVID‐19 infection. (A) Heatmap visualization and clustering analysis of differential cytokines among patients in 0–30 and 61–90 days post‐COVID‐19. (B) Volcano plots depicting cytokines that decrease in 61–90 days group compared with 0–30 days group. Cut off fold change > 1.2, *p* value < 0.05. (C) Relative interleukin levels in the first month (0–30 days) and third month (61–90 days) groups. The data were processed as log10 values. (D) Relative levels of chemokine family members (IP‐10, MCP‐1, MIP1β, and MIP1α) in the first month and third month. The data were processed as log10 values. (E) Relative levels of colony‐stimulating factor (G‐CSF and GM‐CSF) in the first and third month groups. The data were processed as log10 values. (F) Relative levels of interferon (IFN‐γ) in the first and the third month groups. (G) Relative levels of growth factor (PDGF‐BB) in the first and the third month groups. (H) Relative levels of tumor necrosis factor (TNF‐α) in the first and the third month groups. Values are presented as mean ± standard deviation (SD). *p < 0.05, **p < 0.01, and ***p < 0.001 indicated significant difference between the groups.

We first analyzed the changes in cytokine levels in the same individual in the first and second months post‐COVID‐19 (*n* = 20). We observed changes only in IL‐8, IL‐17, G‐CSF, and RANTES levels between the first and second month post‐COVID‐19 (Table S3). Moreover, we analyzed the changes in cytokine levels between the second and third months post‐COVID‐19 (*n* = 38) and found no statistically significant differences (Table S4). We then analyzed the changes in cytokine levels in the same individual between the first and third months post‐COVID‐19 (*n* = 11). Samples obtained in the third month post‐COVID‐19 showed significant differences in cytokine levels compared with samples obtained in the first month (Figure 1A,B). A decrease was observed in the levels of IL‐1β, IL‐6, IL‐7, IL‐8, IL‐9, and IL‐15 (Figure 1C). Additionally, the levels of chemokines, including MIP‐1α, MIP‐1β, IP‐10, and MCP‐1, were reduced (Figure 1D). The levels of CSFs, specifically G‐CSF and GM‐CSF, also exhibited a decline (Figure 1E). Furthermore, reductions in IFN‐γ (Figure 1F), growth factor PDGF‐BB (Figure 1G), and TNF‐α (Figure 1H) levels were noted. The levels of some cytokines did not differ significantly (Table S5). These findings indicate that the levels of these proinflammatory cytokines decreased in the third month, suggesting a moderation in the intensity of the inflammatory response.

### 2.4 Effects of COVID‐19 Infection from the Perspective of Metabolism

**FIGURE 2:**
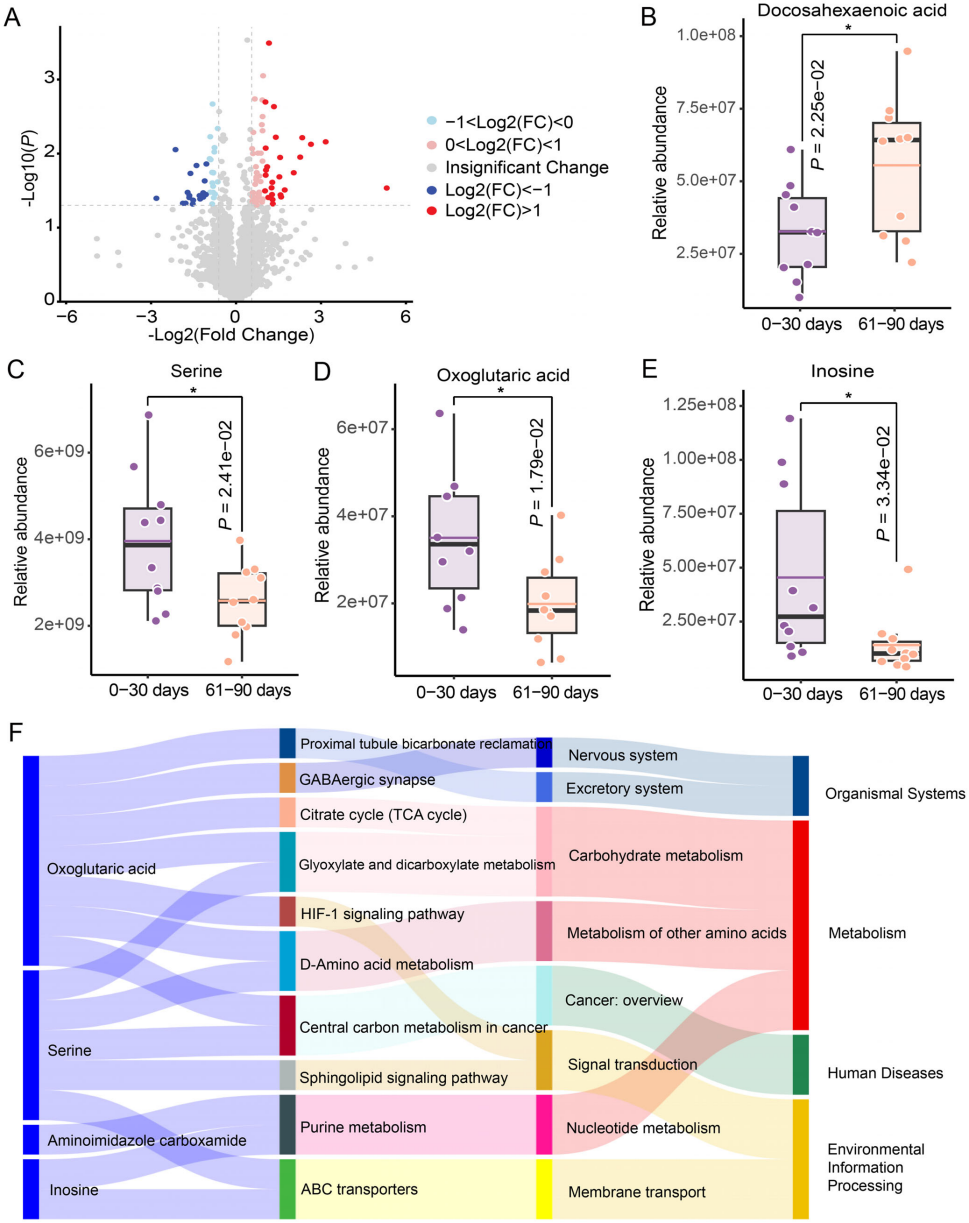
Metabolic profiling of seminal plasma samples at indicated time points post‐COVID‐19 infection. (A) Volcano plots comparing seminal plasma metabolites from patients between 0–30 and 61–90 days post‐COVID‐19. Red represents increased metabolite levels, whereas blue represents decreased metabolite levels. |Fold change| = 1.5, *p* < 0.05. (B–E) Comparison of representative metabolites (docosahexaenoic acid, serine, oxoglutaric acid, and inosine). The colored horizontal lines represent the mean. (F) Sankey diagram illustrating the relationship between the top 10 pathways with significant enrichment and differential metabolites with 0–30 and 61–90 days (*p* < 0.05). Blue represents downregulation.

Recent studies have highlighted the close link between metabolism and immune response [32, 33]. Investigations have also reported the serum metabolic characteristics associated with COVID‐19, reflecting the immune response induced by COVID‐19 infection [34, 35, 36]. To determine whether the inflammation‐induced changes in sperm quality were due to metabolic alterations, we performed an untargeted metabolomic analysis of seminal plasma. Specifically, we examined semen samples from patients post‐COVID‐19, comparing the metabolite profiles at two different time points: 0–30 and 61–90 days postinfection (*n* = 10). The metabolomic analysis identified a total of 2939 metabolites, with 92 metabolites exhibiting significant differences between the two time points. Volcano plot analysis was employed to highlight metabolites with the most pronounced changes (*p* < 0.05, |fold change| = 1.5; 58 up and 34 down) (Figure 2A). Subsequently, Kyoto Encyclopedia of Genes and Genomes pathway enrichment analysis was performed, which revealed 34 pathways that were downregulated and two pathways that were upregulated in infertile men 61–90 days after post‐COVID‐19 compared with 0–30 days. Within the upregulated metabolic pathways, we noted a significant increase in docosahexaenoic acid levels (Figure 2B), potentially facilitating a decline in the levels of inflammatory mediators such as IL‐1β and IL‐6α [37, 38, 39]. Conversely, in the downregulated metabolic pathways, we identified reduced levels of several metabolites, such as serine, oxoglutaric acid, and inosine (Figure 2C–E), which could result in a decrease in inflammatory regulators, including IL‐1β and TNF‐α [40, 41, 42, 43, 44]. Further analysis revealed that these metabolic pathways were primarily associated with purine metabolism (nucleic acid metabolism), D‐amino acid metabolism (amino acid metabolism), and glyoxylate and dicarboxylate metabolism (carbohydrate metabolism) (Figure 2F). These findings suggest that the inflammatory response in patients with COVID‐19 may be associated with disturbances in metabolic homeostasis. Moreover, changes in metabolites or metabolic pathways may play a crucial role in restoring the cytokine balance and modulating inflammatory responses in the body.

### 2.5 Correlation Between Fever and Decline in Sperm Quality

**FIGURE 3:**
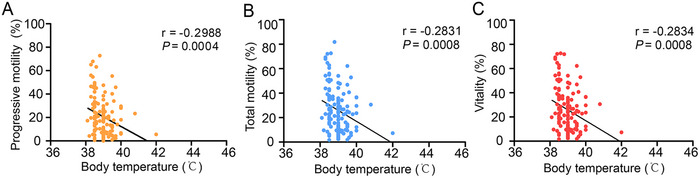
Correlation between semen quality parameters and fever. (A) Spearman's rho correlation of progressive motility (%) with fever at 0–30 days. (B) Spearman's rho correlation of total motility (%) with fever at 0–30 days. (C) Spearman's rho correlation of vitality (%) with fever at 0–30 days.

Previous studies have indicated that fever is the primary symptom associated with COVID‐19 [42, 45, 46], with immune responses often triggering this condition. Additionally, body temperatures above 38°C can affect sperm quality [47]. To investigate the influence of fever on sperm characteristics, we analyzed semen samples from participants who experienced fever with a body temperature exceeding 38°C during COVID‐19. Our findings showed that in the first month post‐COVID‐19 (*n* = 138), when body temperature was above 38°C, vitality, total motility, and progressive motility significantly declined and correlated negatively with body temperature (Figure 3 and Table 3). This suggests that increased body temperature caused by COVID‐19 could hinder sperm quality.

**TABLE 3:** Spearman's rho correlation of semen parameters with fever at different time points.

	0–30 days			31–60 days			61–90 days			≥91 days		
Characteristic	*n*	*r*	*p* value	*n*	*r*	*p* value	*n*	*r*	*p* value	*n*	*r*	*p* value
Volume (mL)	138	−0.1105	0.197	362	0.04924	0.3502	175	−0.01251	0.8695	58	−0.1590	0.2331
pH	138	−0.08993	0.2942	362	0.06389	0.2253	175	0.002973	0.9689	58	−0.07219	0.5902
Sperm concentration (million/mL)	136	−0.1193	0.1665	359	−0.1256	0.0173	175	−0.04651	0.5411	58	0.10460	0.4347
Total sperm count (million)	138	−0.1297	0.1295	362	−0.1063	0.0434	175	−0.03827	0.6151	58	−0.003635	0.9784
Vitality (%)	138	−0.2834	0.0008	362	0.01476	0.7796	175	−0.04281	0.5737	58	−0.1098	0.4121
Progressive motility (%)	138	−0.2988	0.0004	362	−0.0114	0.8289	175	−0.01534	0.8403	58	−0.05872	0.6615
Total motility (%)	138	−0.2831	0.0008	362	0.0136	0.7966	175	−0.02656	0.7271	58	−0.08454	0.5281
Normal morphology (%)	109	−0.1708	0.0759	300	−0.08844	0.1264	112	−0.007257	0.9395	35	0.05102	0.7710
Head defect (%)	109	0.1057	0.2739	300	0.06127	0.2901	112	0.06620	0.488	35	−0.1802	0.3004
Neck defect (%)	109	0.01678	0.8625	300	−0.04998	0.3884	112	0.08877	0.352	35	−0.09993	0.5679
Tail defect (%)	109	−0.003747	0.9692	300	−0.03128	0.5894	112	−0.05528	0.5627	35	0.01671	0.9241

### 2.6 Reproductive Hormone Levels Post‐COVID‐19

Reproductive hormone levels can be used to evaluate male reproductive function, and hormonal levels might be associated with the observed reduction in semen quality after COVID‐19. To investigate this, we examined hormone levels in the serum of 184 patients. All 184 serum samples were analyzed to determine the hormone levels: 22.83% (*n* = 42) in the first month post‐COVID‐19, 44.02% (*n* = 81) in the second month post‐COVID‐19, and 14.13% (*n* = 26) in the third month post‐COVID‐19. Additionally, 19.02% (*n* = 35) of uninfected samples were used as controls. No significant differences in follicle‐stimulating hormone (FSH), luteinizing hormone (LH), testosterone, estradiol, and prolactin levels were found post‐COVID‐19 between the groups at different time points (Table 4). These findings suggest that COVID‐19 may not affect spermatogenesis by altering body hormone levels, but may be associated with the inflammatory response and fever.

**TABLE 4:** Hormonal profiles of male patients who recovered from COVID‐19.

		Time lapse since COVID‐19 infection				*p *value^b^		
Parameter	Pre‐COVID‐19 (*n* = 35)	0–30 days (*n* = 42)	31–60 days (*n* = 81)	61–90 days (*n* = 26)	*p *value^a^	Pre‐COVID‐19 vs. 0–30 days	Pre‐COVID‐19 vs. 31–60 days	Pre‐COVID‐19 vs. 61–90 days
Testosterone (n/mL)	11.11 ± 3.03	9.81 ± 2.48	10.12 ± 2.57	10.28 ± 3.90	0.3765	0.1057	0.1762	0.1970
LH (mIU/mL)	3.96 ± 1.72	4.02 ± 2.19	3.62 ± 1.87	3.85 ± 1.48	0.4574	0.7123	0.1822	0.9107
FSH (mIU/mL)	5.47 ± 2.43	5.57 ± 4.67	4.93 ± 3.62	5.17± 3.24	0.3990	0.2358	0.0787	0.2977
Prolactin (ng/mL)	12.40 ± 8.05	12.56 ± 6.65	12.18 ± 6.85	10.80 ± 5.84	0.4706	0.3736	0.3459	0.7584
Esteradiol (pg/mL)	97.91 ± 23.73	90.10 ± 21.36	93.38 ± 18.07	92.64 ± 25.11	0.4563	0.2161	0.9558	0.6416

Data are presented as mean ± standard deviation. The *p* values of outcome events are calculated using ^a^Kruskal–Wallis test or ^b^Mann–Whitney *U*‐test.

*Abbreviations*: LH, luteinizing hormone; FSH, follicle stimulating hormone.

## 3 Discussion

The impact of SARS‐CoV‐2 on semen parameters is an issue of increasing interest, as it provides biological evidence for clinical recommendations in assisted reproductive medicine centers. Previous studies have reported that COVID‐19 can cause a decline in key semen indicators, including volume, sperm count, and motility [48, 49]. However, these studies have some limitations, such as small sample size, which may lead to sampling error and lack of comparisons at different time points before and after infection. In our study, we collected 1294 semen samples from 781 men with infertility, grouped by different time points after recovery, alongside data on sperm parameters pre‐COVID‐19, which served as controls. Parameters indicative of semen quality, including sperm concentration, vitality, total motility, progressive motility, and sperm morphology, significantly declined in the early post‐COVID‐19. Notably, compared with the controls, the decline in sperm parameters was largely reversed during the third month post‐COVID‐19. By contrary, a study conducted by Cakir et al. [50] suggested that the negative effects of COVID‐19 on semen parameters may persist in the long term. Their findings indicated a significant decrease in semen volume, total sperm count per ejaculate, and total number of sperm with normal morphology in the short‐term and long‐term groups compared with the pre‐COVID‐19 values in the same patients [50]. However, they also noted limitations in their study, including a small sample size and the lack of a statistically normal distribution in the pre‐COVID‐19 semen parameters of the patients in their study [50]. Our study showed that although SARS‐CoV‐2 infection can adversely affect semen quality, this effect is reversible. A plausible explanation is that the spermatogenic cycle only lasts for approximately 74 days [29], suggesting that the impact of COVID‐19 may persist for only one spermatogenic cycle.

Certain viruses, such as Zika virus and human immunodeficiency virus (HIV), can be sexually transmitted via semen [51, 52]. However, a definitive conclusion cannot be drawn regarding the transmission of SARS‐CoV‐2 through semen. A study involving 38 individuals experiencing acute symptoms or in the recovery phase of COVID‐19 found that six semen samples tested positive for SARS‐CoV‐2 [23]. However, this positive result was likely caused by contamination from aerosols or other bodily fluids from patients [53]. Most studies have not detected SARS‐CoV‐2 in the semen [54, 55]. To clarify this, we performed an extensive analysis to detect SARS‐CoV‐2 in semen samples of 96 individuals who had recovered from COVID‐19 at various intervals post‐COVID‐19. Notably, no traces of SARS‐CoV‐2 were found across all time points examined (0–30, 31–60, 61–90, and ≥ 91 days). These findings indicate that SARS‐CoV‐2 RNA is probably absent from semen. Therefore, the observed changes may not be caused by direct SARS‐CoV‐2 infection, and further exploration into other biological mechanisms is warranted.

Additionally, several studies have shown that cytokine storms disrupt the blood–testis barrier in Sertoli cells, leading to reduced sperm concentrations and viability [56]. Available evidence suggests that COVID‐19 can cause autoimmune orchitis in some patients [30]. Moreover, SARS‐CoV‐2 infection in males may result in the upregulation of proinflammatory cytokines (such as IL‐1β, IL‐6, IL‐2, IL‐8, IFN‐γ, and TNF‐α) and stimulate lipid peroxidation in human spermatozoa, thereby impairing sperm viability and survival [26, 57‐59]. Given that cytokine networks are dynamic, it would be valuable to investigate variations in seminal plasma cytokine content over time. In our study, we observed that semen samples collected in the third month post‐COVID‐19 showed relatively lower levels of cytokines, including IL‐1β, IL‐6, IL‐7, IL‐8, IL‐9, IL‐15, G‐CSF, GM‐CSF, IFN‐γ, TNF‐α, PDGF, IP‐10, MIP‐1α, MIP‐1β, and MCP‐1, compared with those in samples in the first month. This indicates a broader range of cytokine alterations, with a general decline in proinflammatory cytokines levels by the third month. The alterations in semen parameters mentioned earlier also indicate that the immune response of the male reproductive system may require an extended period to fully recover following COVID‐19.

In recent years, several studies related to immunometabolism have elucidated the complex relationship between immune responses and metabolism [32, 33]. For example, succinate, a metabolite associated with mitochondrial metabolism, is known to enhance the production of IL‐1β during inflammatory processes [60]. In macrophages, itaconate can stimulate the release of proinflammatory cytokines [61]. Since the onset of the COVID‐19 pandemic, several studies have investigated the immunological and metabolic profiles of infected patients [5, 62‐68]. These studies have demonstrated a strong correlation between serum metabolites and inflammatory cytokines in patients with COVID‐19 [65, 66, 67]. Studies have found that metabolites are closely linked to cytokines (IL‐6, IL‐1α, IL‐1β, and M‐CSF), and interventions in the metabolism of arginine, tryptophan, or purines can effectively modulate the release of proinflammatory cytokines in peripheral blood mononuclear cells infected with SARS‐CoV‐2 [65, 67]. In this study, we observed significant alterations in metabolite levels in semen samples from patients 61–90 days after COVID‐19 compared with 0–30 days. Notably, these changes were associated with pathways related to purine metabolism, D‐amino acid metabolism, and glyoxylate and dicarboxylate metabolism. Studies have shown that these metabolites or metabolic pathways can modulate the expression and secretion of cytokines through diverse mechanisms, which in turn can influence immune responses and inflammatory processes. In a study of extremely preterm infants, docosahexaenoic acid levels were inversely correlated with inflammation‐related proteins such as IL‐6 and CCL7 [37]. In lipopolysaccharide (LPS)‐stimulated macrophages, docosahexaenoic acid effectively suppresses the production of proinflammatory cytokines such as IL‐1β and IL‐6 [38]. In glial cells, docosahexaenoic acid reduces the secretion of TNF‐α and IL‐6 by inhibiting the activation of NF‐κB and AP‐1 transcription factors [39]. Serine plays a crucial role in the LPS‐induced expression of IL‐1β mRNA expression and inhibiting de novo serine synthesis in vivo reduces LPS‐induced IL‐1β levels while improving survival in a mouse sepsis model [40, 41]. In the urine metabolites from patients with COVID‐19, oxoglutaric acid was found to be significantly associated with changes in IL‐4 levels, suggesting its potential role in regulating cytokine networks during the inflammatory response [43]. In non‐human primate models infected with HIV‐1/simian immunodeficiency virus, elevated inosine levels were associated with markers of immune activation and disease progression [44]. In certain cases, inosine can enhance the production of IL‐1β, particularly when stimulated by the NLRP3 inflammasome [69]. These findings imply that the inflammatory response of patients with COVID‐19 is related to the disruptions in metabolic homeostasis, including amino acid metabolism, nucleic acid metabolism, and carbohydrate metabolism. The interplay between metabolites and cytokines is not merely a linear relationship but rather a complex, bidirectional regulatory network, the detailed mechanisms of which still require further exploration.

Intense immune responses are often accompanied by fever, which is a common symptom of COVID‐19, and spermatogenesis is temperature dependent. Several studies have found that sperm concentration, sperm count, and motile sperm rate in individuals who recovered from COVID‐19 and had fever during the illness were lower than those in individuals without fever. However, these findings were based on a limited sample size [70]. Temiz et al. [45] showed significant changes in normal semen morphology in patients with COVID‐19 and hypothesized that this was caused by fever. Nonetheless, another study found that sperm motility and viability are negatively affected in individuals with COVID‐19, irrespective of whether they experience fever [71]. These inconsistent results may stem from limited sample sizes or possible differences in the degree of fever among the populations recruited in these studies. Our study demonstrated a link between elevated body temperature exceeding 38°C during COVID‐19 and sperm quality indicators. We observed that during the initial month post‐COVID‐19 (*n* = 138), when body temperature was above 38°C, three parameters (vitality, progressive motility, and total motility) showed a negative correlation with body temperature, suggesting that fever can affect semen parameters.

SARS‐CoV‐2 can cross the blood–brain barrier and promote neuroinflammation in the brain upon infection ACE2‐expressing cells, resulting in physiological dysfunction in body temperature regulation and hormone balance [72]. Therefore, the cause of decreased sperm quality in patients with COVID‐19 may be dysfunction of the hypothalamic‐pituitary‐testicular axis, which leads to abnormal secretion of gonadotropin‐releasing hormone, LH, and FSH, affecting testosterone production and spermatogenesis. Some studies have reported an increase in LH levels in individuals infected with SARS‐CoV‐2, along with a decrease in the testosterone/LH and FSH/LH ratios. Contrary to these findings, no significant difference was observed in testosterone levels between men with COVID‐19 and uninfected men [73]. Our study revealed that testosterone levels in all samples at different time points post‐COVID‐19 remained within normal range. Additionally, the levels of FSH, LH, estradiol, and prolactin were comparable to those in the pre‐COVID‐19 group, with no significant differences observed. These findings align with the results of the study conducted by Guo et al [74]. It is necessary to expand the sample size to study the correlation between testosterone levels and COVID‐19 and to explore the effects of hormone levels on male reproduction. Our results suggest that COVID‐19 infection may not affect spermatogenesis by altering hormone levels but may be associated with inflammatory responses and fever.

This study has some limitations. We lacked semen samples and data on cytokine levels pre‐COVID‐19 infection. We have only explored the potential mechanism from a metabolic perspective, but the specific molecular mechanism has not yet been elucidated. Further research is needed to explore the disrupted pathways and identify specific treatment methods.

In conclusion, we comprehensively assessed sperm quality in individuals diagnosed with infertility after recovery from COVID‐19. No evidence was found to suggest that SARS‐CoV‐2 is sexually transmitted through semen following COVID‐19, as no viral RNA was detected in any of the examined semen samples. Critical indicators of sperm quality, including sperm concentration, vitality, total motility, progressive motility, and sperm morphology, were significantly compromised in the first month post‐COVID‐19. However, by the third month post‐COVID, these parameters largely returned to preinfection levels. The decline in sperm quality was associated with inflammatory responses and fever but not with hormone levels. These findings provide valuable insights for fertility specialists and help refine the guidance provided to male patients with infertility who are considering assisted reproductive technology.

## 4 Methods and Materials

### 4.1 Study Design and Participants

This study involved 781 male participants, aged 20–55 years, who were recruited between January 12, 2023 and April 4, 2023, at the Reproductive Medicine Center of Peking University Third Hospital. All participants had not used any contraceptive measures for more than 12 months and had a normal sex life without a successful pregnancy. During the initial medical evaluation, specific sperm abnormalities were identified according to World Health Organization's (WHO) criteria. These conditions included a sperm concentration of less than 15 × 10^6^ spermatozoa/mL (oligozoospermia), a progressive sperm motility rate below 32% (asthenozoospermia), and a sperm morphology with less than 4% normal forms (teratozoospermia). All participants were confirmed to have an initial infection through pharyngeal swab tests using the reverse transcription‐PCR (RT‐PCR) or antibody detection method at the reproductive medicine center or community hospitals, and they all recovered without hospital‐based interventions. Participants with other acute or severe chronic diseases were also excluded.

### 4.2 Sperm Sampling and Preparation

Semen samples were collected from each participant through masturbation. The participants were asked to abstain from sexual activity for 3–6 days. Freshly collected semen samples were liquefied at room temperature for 30–60 min and processed within 1 h of collection. Semen samples collected for analysis were categorized according to the time elapsed since recovery from COVID‐19, allowing for a comparative study across various time intervals: samples were gathered in the first month postinfection (0–30 days), the second month (31–60 days), the third month (61–90 days), and beyond the third month (≥91 days). Additionally, a control group was established using data from infertile men before COVID‐19 infection.

### 4.3 Detection of SARS‐CoV‐2 in the Semen Samples

The samples were processed in the RT‐PCR laboratory at Peking University Third Hospital. The laboratory team had extensive experience in detecting SARS‐CoV‐2 in clinical samples. SARS‐CoV‐2 was detected by RT‐PCR using the SARS‐CoV‐2 RNA assay kit (Sansure Biotech Inc., Hunan, China; National Instrument Standards number for the test kit is 20203400064), strictly following the manufacturer's protocol.

### 4.4 Analysis of Semen Quality Parameters

All analyses were performed by an experienced technician blinded to the study. Following the standards of the WHO, semen samples were comprehensively evaluated for a range of key parameters using a computer‐assisted sperm analysis system (SSA‐II, Suijia Software Co. Ltd, Beijing, China). The parameters assessed included semen volume (mL), total sperm count (million), sperm concentration (10^6^/mL), vitality (%), total motility (%), progressive motility (%), and the percentage of sperm cells with normal morphology (%).

### 4.5 Cytokine Assays

Semen samples were centrifuged, frozen at −80°C, and analyzed simultaneously. Following the manufacturer's guidelines, cytokine levels in semen samples were quantified using 27‐plex cytokine assay kits from Wayen Biotechnologies (Shanghai, China). The following analytes were measured: IL‐1β, IL‐1ra, IL‐2, IL‐4, IL‐5, IL‐6, IL‐7, IL‐8, IL‐9, IL‐10, IL‐12, IL‐13, IL‐15, IL‐17, G‐CSF, GM‐CSF, IFN‐γ, TNF‐α, PDGF‐BB, FGF, VEGF, IP‐10, eotaxin, MIP‐1α, MIP‐1β, RANTES, and MCP‐1.

### 4.6 Untargeted Metabolomics

Each semen sample (100 µL) was mixed with 300 µL of precooled methanol and incubated at −20°C for 30 min. The mixture was then centrifuged at 16,000×*g* for 20 min at 4°C to obtain the supernatant, which was used for mass spectrometry analysis. Chromatographic separation was performed by SHIMADZU‐LC30 Ultra high performance liquid chromatography system and ACQUITY UPLC HSS T3 (2.1 × 100 mm, 1.8 µm) chromatographic column. The samples were then analyzed by mass spectrometry using the QE Plus mass spectrometer (Thermo Scientific). The MSDIAL software was used for peak alignment, retention time correction, and peak area extraction of the raw data. Python software was used for pattern recognition on the extracted data. For preprocessing, unit variance scaling was applied, followed by further data analysis.

### 4.7 Evaluation of Reproductive Hormone Levels

Serum samples were obtained from the peripheral blood samples of the participants. Serum concentrations of testosterone, LH, FSH, estradiol, and prolactin were assessed using chemiluminescent immunoassay kits (L2KFS6, L2KLH6, L2KTW6, L2KE22, and L2KPR6) and the IMMULITE 2000 XPi Instruments from SIEMENS.

### 4.8 Statistical Analysis

Data were analyzed using GraphPad Prism Version 8. Statistical significance was evaluated using the *t*‐test, non‐normally distributed data were evaluated using the Mann–Whitney *U*‐test, differences across multiple groups were evaluated using the Kruskal–Wallis test, and the correlation between variables was analyzed using Spearman's Rho. Data were considered significant when the *p* value was less than 0.05 (*), 0.01 (**), or 0.001 (***).

## Author Contributions

This project was conceived and coordinated by K. H., L. Y., J. Q., and Q. L. Y. Z. and F. Z. performed most of the acquisition and analysis of data under the supervision of Q. L., Z. Z., and K. H. Z. Z. and Y. X. provided assistance for sample collection. J. W. and T. L. assisted in data analysis. Z. Z., P. Y., D. L., H. Z., H. L., J. M., W. T., and K. H. collected the patient information. Y. Z., F. Z., and Q. L. prepared the manuscript. All authors discussed the results and commented on the manuscript. All authors have read and approved the final manuscript.

## Ethics Statement

The study protocol was reviewed and approved by the Ethics Committee of Peking University Third Hospital (Reg. no. IRB00006761‐M2023176). Informed consent was obtained from all patients at the time of enrolment.

## Conflicts of Interest

The authors declare no conflicts of interest.

## Supporting information



Supporting Information

## Data Availability

The data underlying this article will be shared upon reasonable request by the corresponding author.
