# Supplementary material for: Alterations in Semen Quality and Immune‐Related Factors in Men with Infertility who Recovered from COVID‐19
Source: MedComm (2020). 2025 Apr 24;6(5):e70179. doi: 10.1002/mco2.70179 (PMC12019875; doi:10.1002/mco2.70179)
Supplement: Supplementary file 1 — Supporting Information [file MCO2-6-e70179-s001.pdf]

Supplementary Information

For

Alterations in semen quality and immune-related factors in men with infertility who recovered from COVID-19

Ying Zhang[1,2#], Feiyin Zhu[1,2,3#], Zhe Zhang[1,4], Jing Wang[1,2], Tianyi Liao[1,2,3], Yu Xi[1,4], Defeng Liu[1,4], Haitao Zhang[1,4], Haocheng Lin[1,4], Jiaming Mao[1,4], Wenhao Tang[1,4], Lianming Zhao[1,4], Peng Yuan[1,2], Liying Yan[1,2], Qiang Liu[1,2*], Kai Hong[1,4*], Jie Qiao[1,2,3]

[1]Center for Reproductive Medicine, Department of Obstetrics and Gynecology, Peking University Third Hospital, Beijing, China.

[2]State Key Laboratory of Female Fertility Promotion, National Clinical Research Center for Obstetrics and Gynecology, Key Laboratory of Assisted Reproduction (Peking University), Ministry of Education, Beijing Key Laboratory of Reproductive Endocrinology and Assisted Reproductive Technology, Peking University Third Hospital, Beijing, China

[3]Peking-Tsinghua Center for Life Sciences, Peking University, Beijing, China.

[4]Department of Urology, Peking University Third Hospital, Beijing, China

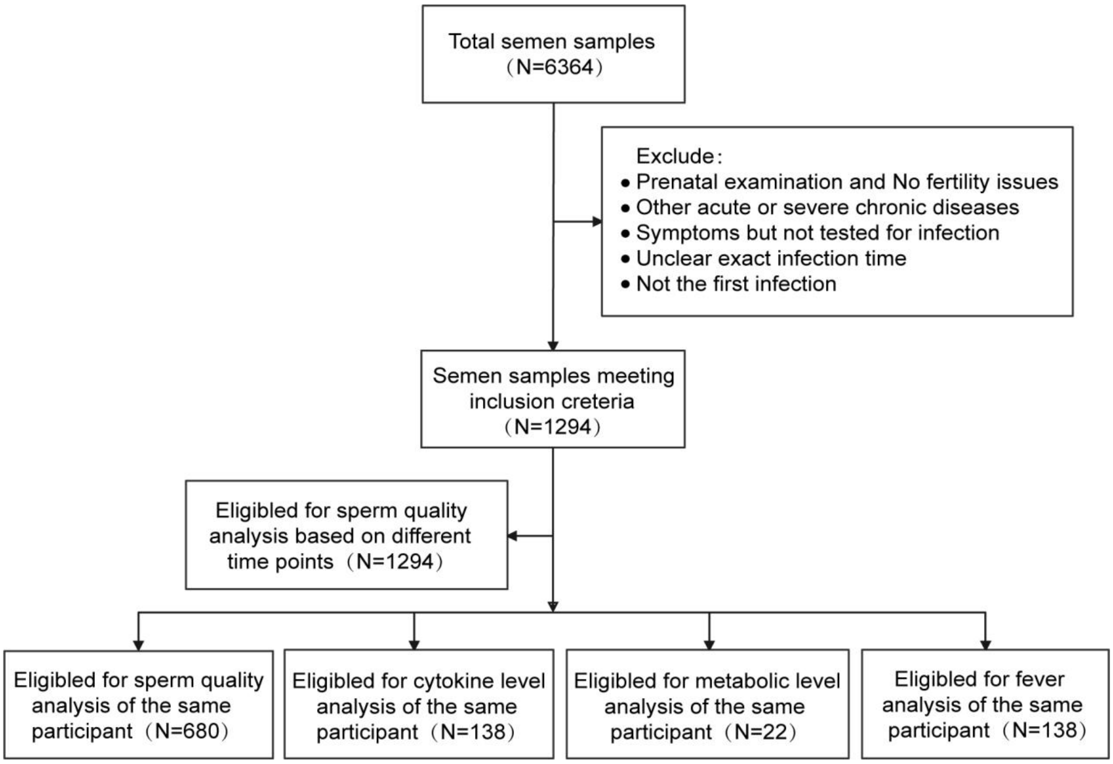

Supplementary Figure 1. Flow chart for study selection and inclusion. N represents the number of samples.

Supplementary Table 1. Sperm quality at different time points post-COVID-19

		**Time lapse since COVID-19 infection**		***P* value**	
**Characteristic**	**Pre-COVID-19**	**0–30 days**	**61–90 days**	**Pre-COVID-19 vs. 0–30 days**	**Pre-COVID-19 vs. 61–90 days**
No. of samples	234	203	248	/	/
Volume, mL	3.38 ± 2.39	3.03 ± 1.31	3.16 ± 1.28	0.1080	0.3635
Sperm concentration, million/mL	46.75 ± 35.86	32.22 ± 29.55	47.38 ± 42.55	<0.0001	0.8562
Total sperm count, million	141.92 ± 123.72	98.15 ± 85.00	136.12 ± 107.07	<0.0001	0.6646
Vitality, %	33.88 ± 18.89	27.29 ±17.31	34.66 ± 18.54	0.0002	0.6199
Progressive motility, %	28.30 ± 17.93	21.9 ± 15.88	28.73 ± 16.94	<0.0001	0.6296
Total motility, %	33.89 ± 18.88	27.34 ± 17.45	34.41 ± 18.34	0.0002	0.7058
Normal morphology, %	2.45 ± 1.21	2.00 ± 1.29	2.73 ± 1.51	0.0002	0.1807
Head defect,%	97.02 ± 9.37	96.97 ± 1.82	96.00 ± 2.48	0.0009	0.5975
Neck defect,%	0.63 ± 0.51	0.59 ± 0.60	0.69 ± 0.66	0.7240	0.1142
Tail defect,%	0.55 ± 0.44	0.43 ± 0.53	0.50 ± 0.60	0.0413	0.4813

Data are presented as mean ± standard deviation. The *P* values of outcome events are calculated using Mann–Whitney U test. / means not available.

Supplementary Table 2. Detection of SARS-CoV-2 RNA in the semen at the indicated times post-COVID-19

	Time lapse since COVID-19 infection			
**Characteristic**	**0–30 days**	**31–60 days**	**61–90 days**	**≥91 days**
No. of participants	33 (34.38%)	46 (47.92%)	12 (12.50%)	5 (5.21%)
No. of days postinfection [range]	18.00 ± 10.07 [1-29]	44.51 ± 7.94 [31-60]	69.26 ± 6.84 [63-81]	102.20 ± 9.63 [91-116]
SARS-CoV-2 RNA in semen	negative	negative	negative	negative

Data are presented as mean ± standard deviation [range] or number (%).

Supplementary Table 3. Differences in cytokine levels between the first- (0–30 days) and second-month (31–60 days) groups post-COVID-19

	**Time lapse since COVID-19 infection (n = 20)**		
**Cytokine**	**0–30 days**	**31–60 days**	***P* value**
IL-1β	1.61 ±1.49	1.17 ± 0.28	0.5892
IL-1ra	46.95 ± 43.00	41.03 ± 45.16	0.6686
IL-2	1.78 ±1.04	1.59 ± 0.97	0.3743
IL-4	2.53 ± 0.63	2.48 ± 0.52	0.7302
IL-5	99.59 ± 22.99	97.37 ± 27.13	0.7424
IL-6	18.89 ± 22.65	12.62 ± 13.04	0.3683
IL-7	719.46 ± 382.46	714.43 ± 353.23	0.6215
IL-8	295.65 ± 318.45	195.17 ± 126.08	0.0266*
IL-9	71.04 ± 23.86	69.75 ± 18.15	0.5949
IL-10	2.60 ± 3.93	1.36 ± 1.77	0.1230
IL-12 (p70)	4.62 ± 5.42	4.02 ± 4.01	0.7736
IL-13	0.25 ± 0.79	0.10 ± 0.11	0.9453
IL-15	18.92 ± 12.39	18.43 ± 10.72	0.8306
IL-17	3.66 ± 1.87	2.86 ± 0.64	0.0369*
G-CSF	75.47 ± 130.03	34.32 ± 56.96	0.0064**
GM-CSF	0.80 ± 0.58	0.67 ± 0.52	0.0795
IFN-γ	20.08 ± 9.71	17.85 ± 10.62	0.3104
TNF-α	53.74 ± 21.50	41.95 ± 19.44	0.0976
PDGF-BB	626.77 ± 365.07	618.93 ± 282.25	0.9058
Basic.FGF	4.68 ± 1.22	4.38 ± 0.76	0.1114
VEGF	2228097.44 ± 2107452.79	2458355.97 ± 2143195.56	0.3828
IP-10	68840.48 ± 15935.32	72635.30 ± 21945.09	0.2743
MIP-1α	3.86 ± 7.04	1.63 ± 1.74	0.3300
MIP-1β	82.47 ± 82.95	55.25 ± 38.56	0.2024
RANTES	86.46 ± 46.70	117.25 ± 67.83	0.0012**
Eotaxin	40.18 ± 13.76	45.73 ± 19.34	0.5217
MCP-1	746.62 ± 490.65	649.42 ± 358.23	0.2664

Data are presented as mean ± standard deviation. The *P* values of outcome events are calculated using paired t-test. $*P < 0.05$, $**P < 0.01$. IL, interleukin; G-CSF, granulocyte-colony stimulating factor; GM-CSF, granulocyte macrophage-colony stimulating factor; IFN-γ, interferon-gamma; TNF-α, tissue necrosis factor alpha; PDGF, platelet-derived growth factor; FGF, fibroblast growth factor; VEGF, vascular endothelial factor; MIP, macrophage inflammatory protein; MCP, monocyte chemoattractant protein.

Supplementary Table 4. Differences in cytokine levels between the second- (31–60 days) and third-month (61–90 days) groups post-COVID-19

	**Time lapse since COVID-19 infection (n = 38)**		
**Cytokine**	**31–60 days**	**61–90 days**	***P* value**
IL-1β	1.41 ± 2.10	2.50 ± 9.16	0.6199
IL-1ra	55.00 ± 69.00	42.35 ± 25.41	0.4358
IL-2	1.69 ± 0.77	1.52 ± 0.68	0.1049
IL-4	2.22 ± 0.36	2.20 ± 0.39	0.7086
IL-5	101.91 ± 21.24	105.72 ± 24.41	0.2743
IL-6	5.60 ± 4.24	6.04 ± 5.64	0.7856
IL-7	625.67 ± 361.83	673.74 ± 378.02	0.2551
IL-8	124.58 ± 75.54	126.99 ± 70.68	0.7308
IL-9	63.68 ± 13.61	61.30 ± 14.16	0.4210
IL-10	1.67 ± 1.66	1.38 ± 1.82	0.1283
IL-12 (p70)	2.77 ± 2.86	2.74 ± 2.73	0.8755
IL-13	0.08 ± 0.08	0.08 ± 0.09	0.9630
IL-15	23.46 ± 13.74	21.92 ± 12.96	0.4802
IL-17	2.92 ± 0.79	2.79 ± 0.82	0.3257
G-CSF	20.59 ± 25.39	22.68 ± 29.72	0.5975
GM-CSF	0.96 ± 0.56	0.94 ± 0.50	0.7838
IFN-γ	13.63 ± 9.99	13.69 ± 7.85	0.8610
TNF-α	36.70 ± 11.43	34.74 ± 15.27	0.2687
PDGF-BB	542.18 ± 355.30	604.46 ± 375.48	0.0512
Basic.FGF	4.53 ± 1.24	4.80 ± 1.10	0.1155
VEGF	1532450.89 ± 1552494.83	1485222.97 ± 1444577.65	0.8501
IP-10	60267.24 ± 14929.63	61211.86 ± 16095.67	0.9429
MIP-1α	1.15 ± 1.48	1.33 ± 2.62	0.6130
MIP-1β	44.03 ± 27.59	47.96 ± 62.01	0.2670
RANTES	114.48 ± 92.46	111.66 ± 76.00	0.9990
Eotaxin	40.62 ± 14.23	40.59 ± 14.81	0.9771
MCP-1	615.25 ± 371.32	641.30 ± 381.45	0.3965

Data are presented as mean ± standard deviation. The *P* values of outcome events are calculated using paired t-test.

Supplementary Table 5. Differences in cytokine levels between the first- (0–30 days) and third-month (61–90 days) groups post-COVID-19

	Time lapse since COVID-19 infection (n = 11)		
**Cytokine**	**0–30 days**	**61–90 days**	***P* value**
IL-1ra	40.46 ± 20.76	44.09 ± 13.29	0.1855
IL-2	2.80 ± 0.90	2.46 ± 0.52	0.2406
IL-4	3.29 ± 0.68	3.50 ± 0.51	0.0986
IL-5	158.40 ± 27.88	173.94 ± 36.04	0.1350
IL-10	1.95 ± 0.63	1.52 ± 0.68	0.0736
IL-12 (p70)	5.79 ± 2.98	5.56 ± 2.47	0.8215
IL-13	0.08 ± 0.03	0.09 ± 0.07	0.9999
IL-17	4.03 ± 0.88	4.41 ± 1.01	0.1563
Basic.FGF	7.76 ± 1.07	7.47 ± 1.19	0.3125
VEGF	1126414.81 ± 540109.76	1310842.89 ± 419159.96	0.5000
RANTES	110.14 ± 55.89	118.24 ± 54.91	0.0527
Eotaxin	53.52 ± 14.08	48.60 ± 19.03	0.2147

Data are presented as mean ± standard deviation. The *P* values of outcome events are calculated using paired t-test.
